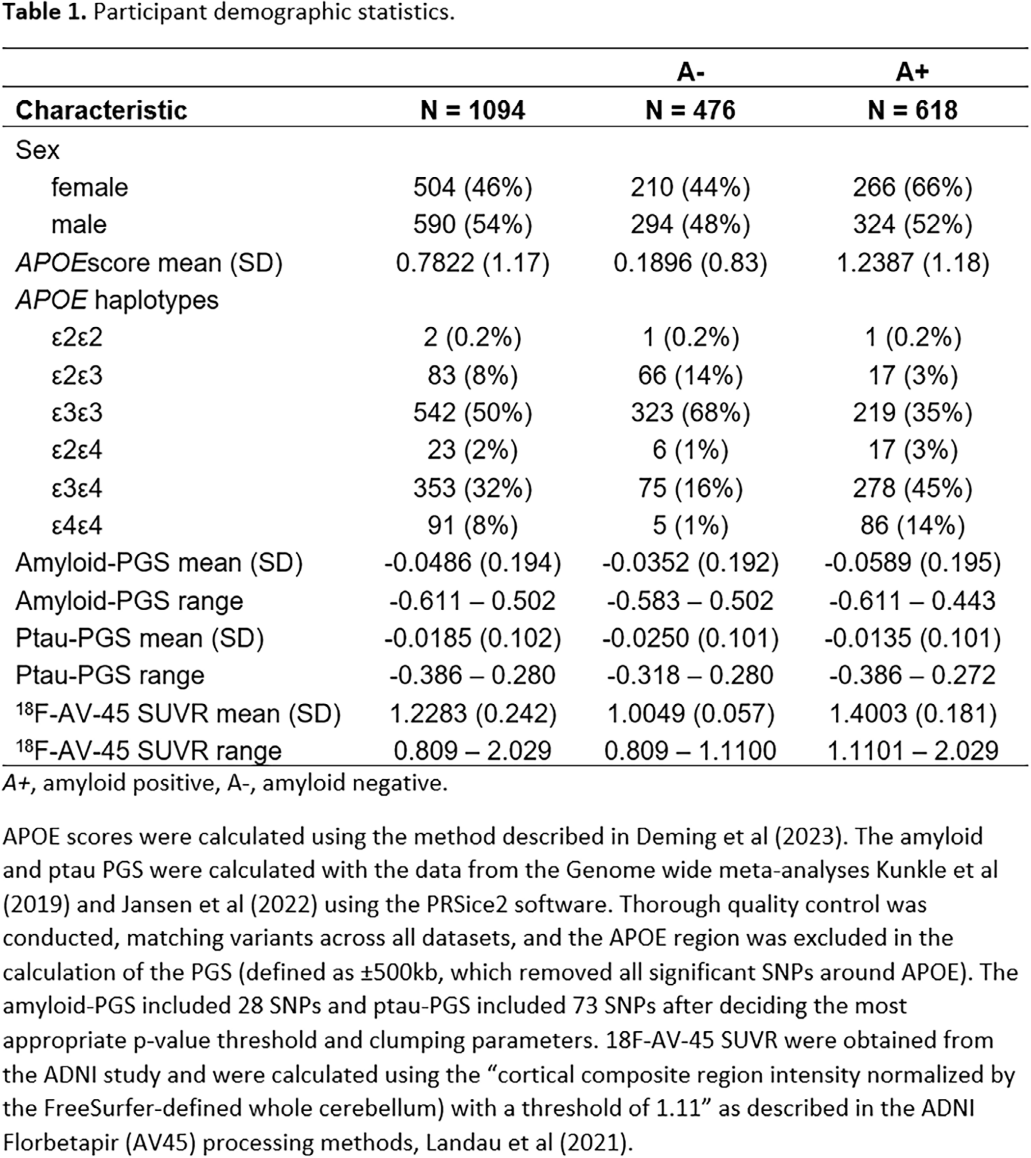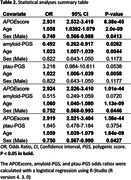# Alzheimer’s disease *APOE* neuropathology‐based score, amyloid, and ptau polygenic scores’ association with amyloid PET positivity

**DOI:** 10.1002/alz.086164

**Published:** 2025-01-03

**Authors:** Yazan Hammad, Yuetiva Deming, Jacob Morse, Ethan Grover, Barbara B. Bendlin, Tobey J. Betthauser

**Affiliations:** ^1^ Wisconsin Alzheimer’s Disease Research Center, University of Wisconsin School of Medicine and Public Health, Madison, WI USA; ^2^ Department of Medical Physics, University of Wisconsin‐Madison, Madison, WI USA

## Abstract

**Background:**

*APOE* is the greatest genetic risk factor for AD, however, other smaller genetic effects are often ignored. In this work, endophenotype‐informed polygenic scores (PGS) that exclude the *APOE* region were tested along with a separate, previously published, *APOE* neuropathology‐based score (*APOE*score). The *APOE*score serves as a more nuanced quantification of *APOE* genetic risk that considers the effects of the different haplotypes. PGS and *APOE*score were compared to amyloid positivity, determined via PET imaging, which is used as a measure for AD risk and progression.

**Methods:**

Alzheimer’s Disease Neuroimaging Initiative (ADNI) participants with genetic data and Florbetapir (^18^F‐AV‐45) amyloid PET summary SUVR (whole cerebellum reference region) values were included in analyses. Amyloid positivity (A+) was defined as SUVR > 1.11. PGS were calculated using the weights from genome‐wide association studies (GWAS) of CSF Aβ_42_ (amyloid‐PGS) or ptau_181_ (ptau‐PGS), after excluding the *APOE* region (±500kb). *APOE* effects were accounted for using the *APOE*score that was calculated following Deming et al (2023).

**Results:**

1094 participants (618 A+) were included in the analyses (Table 1). The *APOE*score was significantly associated with A+ after accounting for sex and age at scan (OR = 2.931, P = 6.38e‐45; Table 2). The amyloid‐PGS was also significantly associated with A+ (OR = 0.492, P = 0.026), whereas the ptau‐PGS did not quite reach significance (OR = 3.218, P = 0.054). After adding the *APOE*score, the effect of amyloid‐PGS on A+ remained but did not quite reach significance (OR = 0.515, P = 0.072) and ptau‐PGS was not significant (OR = 1.845, P = 0.375).

**Conclusion:**

These results validate the utility of the *APOE*score with PET amyloid as well as the potential value of including non‐*APOE* genes in quantifying the genetic risk for amyloid accumulation and AD. The significant association between amyloid‐PGS and A+, and near significance after adding the *APOE*score, demonstrates the existence of additional genetic effects outside of *APOE* that have an impact on amyloid accumulation. While the ptau‐PGS did not reach significance, possibly due to sample size, the OR (3.218) was greater in magnitude than the amyloid‐PGS (1/OR = 2.033). Future work will explore these relationships in other cohorts and with other PET AD biomarkers.